# Impact of Oral Administration of *Lactobacillus reuteri* LMG-P 27481 on Human Gut Microbiota Diversity and Function: A Pilot Study

**DOI:** 10.3390/biomedicines13112840

**Published:** 2025-11-20

**Authors:** Veronica Ojetti, Carmine Petruzziello, Alessio Migneco, Marcello Candelli, Angela Saviano

**Affiliations:** 1 Unicamillus University, 00131 Rome, Italy; 2 Emergency Department, San Carlo di Nancy Hospital, 00165 Rome, Italy; cpetruzziello@gvmnet.it; 3 Emergency Department, Fondazione Policlinico Agostino Gemelli, IRCCS, 00168 Rome, Italy; alessio.migneco@policlinicogemelli.it (A.M.); marcello.candelli@policlinicogemelli.it (M.C.); angela.saviano@policlinicogemelli.it (A.S.)

**Keywords:** *L. reuteri* LMG-P 27481, gut microbiota, colonoscopy, abdominal pain, diarrhea, constipation, probiotics

## Abstract

**Background**: Many literature studies have reported the beneficial effects of probiotics on human health, but few articles have evaluated their “real effects” on the modulation of microbiota after their use. *Lactobacillus reuteri* (*L. reuteri*) is one of the most studied probiotics with the best effects on gut microbiota. **Aims**: The primary aim of our study was the evaluation of the intestinal colonization by *L. reuteri*-LMG P 27481 and its effects on the modification of the gut bacterial flora. The secondary aim was the evaluation of side effects through the validated Gastrointestinal Symptom Rating Scale (GSRS). **Patients and Methods**: This is an interventional, open-label study conducted on 20 healthy adults (10 men and 10 women M/F; mean age 34 ±15 years) who received a probiotic Reuterin^®^ LMG (*L. reuteri* LMG P 27481) for 28 consecutive days in drops at a concentration of 1 × 10^9^ (five drops per day). Microbiota analysis was performed at enrollment (T0), at the end of probiotic supplementation (T1) and after a 14-day follow-up period (T2). **Results**: In our study we observed interesting quantitative and functional variations as regards the Firmicutes/Bacterioidetes ratio, intestinal permeability, and the production of short-chain fatty acids (SCFA). This probiotic was safe and was able to improve patients’ symptoms. **Conclusions**: The intake of *L. reuteri* LMG-P 27481 in healthy subjects showed transitory variations in some functional and metabolic gut functions, especially an improvement in the barrier effect and intestinal permeability, y and an increase in SCFA. Future studies should include target populations to have a greater range for modulation of the gut microbiota.

## 1. Introduction

The gut microbiota influences host health, regulating digestive processes as well as metabolic, immune, and neurological functions [1]. The gut microbiota can be influenced by probiotics, which are live microorganisms studied for their positive impact on metabolic and immune activities [2]. Probiotics favor the heterogeneity of gut communities and prevent the disruption of microbial balance (a condition known as dysbiosis) through many mechanisms, such as the stimulation of beneficial bacteria, the inhibition of harmful strains, the reduction of gut permeability, and the improvement of chronic inflammation [3]. Currently, some of the mechanisms underlying the effects of probiotics remain unclear [4]. Firstly, probiotics enhance the production of short-chain fatty acids (SCFAs) such as acetate, propionate, and butyrate [5]. Butyrate promotes the maintenance of the intestinal barrier, reducing intestinal permeability [6] and oxidative stress [7] by strengthening tight junction proteins (occludins and claudins). Moreover, it promotes the activity of regulatory T-cells (Treg), leading to a decrease of pro-inflammatory cytokines. Furthermore, it influences gut–brain communication through the production of neurotransmitters [8]. Probiotics de-conjugate bile salts, and reduce the absorption of cholesterol and the associated cardiovascular risk [9]. Literature studies demonstrate a key role of probiotics in maintaining intestinal barrier integrity, regulating immune function, and influencing host metabolism [10]. The efficacy of probiotics can be influenced by some host factors (age, diet, host health, etc.), the specific strains used, administration in the form of fermented foods or supplements, the variability among individuals, and their resistance to gastrointestinal transit [11]. Probiotics are mainly classified into *Lactobacillus* sp., *Bifidobacterium* sp., and *Saccharomyces* sp. [12]. Two of the most studied probiotics are *Lactobacillus rhamnosus* (*L. rhamnosus*) and *Lactobacillus reuteri* (*L. reuteri*). These probiotics are able to enhance the production of mucin, strength the tight junctions, promote immune T-reg cell responses, and increase the release of anti-inflammatory cytokines such as interleukin-10 (IL-10) [13]. *L*. *reuteri* LMG P 27481 is a specific strain with some very interesting properties. The genetic analysis of *L. reuteri* highlighted the presence of 188 exclusive genes that provided its specific properties. The results have been confirmed by studies conducted both in vitro and on animal models, with evidence of powerful anti-inflammatory activity. *L.reuteri* stimulates the release of IL-10 and exerts antagonistic activities against the pathogenic *Clostridioides difficile*. In vitro analysis of the novel strain *L. reuteri* LMG P-27481 demonstrated promising results for both safety—including antibiotic sensitivity and genomic profile—and putative efficacy, encompassing resistance to gastrointestinal transit, adhesiveness, cytokine induction, and the release of antimicrobial metabolites [14]. Analysis of the gut microbiota after the oral assumption of probiotics reveals how these beneficial bacteria interact with the existing human gut microbial community, with a variable impact. Some studies confirm that *Lactobacillus* spp. and *Bifidobacterium* spp. strains significantly modified the gut microbiota composition, increasing beneficial bacteria and decreasing harmful species. Klemenak et al. [15] found that probiotics positively influenced the gut microbiota of healthy subjects, increasing the abundance of beneficial strains and, at the same time, reducing pathogenic bacteria. Kristensen et al. [16] conducted a systematic review to examine the impact of probiotic supplementation on the fecal microbiota of healthy subjects. They analyzed 7 RCTs and concluded that probiotics have no effect on the composition of the human gut. We conducted an open-label trial in which 20 healthy adults took this probiotic strain for 28 days, with the aim of assessing the beneficial effects of *L. reuteri* LMG P-27481 and better defining its impact on human gut microbiota composition, functions, and gastrointestinal symptoms.

## 2. Study Objectives

The primary objective of this study was the assessment of modifications in gut microbiota composition through the analysis of fecal microbiota from stool samples collected at enrollment (T0), after 28 days of treatment (T1), and after 14 days of follow-up (T2). The secondary objective is to assess any side effects and modification of GI symptoms using a validated scale, the Gastrointestinal Symptom Rating Scale (GSRS) [17].

## 3. Patients and Methods

We conducted this single-center, open-label controlled trial on 20 healthy adult patients (10 M/10 F, mean age 34.5 ± 15.5 years) who are employed in the San Carlo di Nancy GVM Care and Research Hospital in Rome, between October 2024 and March 2025.

The inclusion criteria were:

- Age > 18 yrs;
- No comorbidity
- No home drug therapies
- Signed informed consent

The exclusion criteria were:

- Age < 18 yrs;
- Pregnant or breastfeeding women
- Documented intake of probiotics or antibiotics during the last 30 days
- Intake of home drug therapies
- Cardiovascular, pulmonary, renal, gastrointestinal, and neoplastic comorbidities
- Inclusion in another clinical trial that ended less than 7–10 days earlier
- Previous abdominal surgical intervention

**Figure 1:**
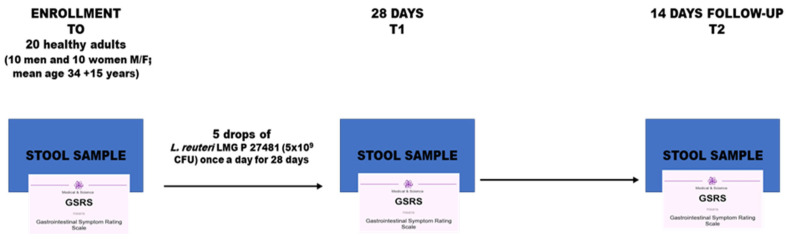
Flow Chart of the study.

Patients could withdraw from the study at any time (according to personal choice) or in case of adverse effects related to the administered drugs. At enrollment, a physician collected demographic data from all patients and performed a physical examination. All patients received the Gastrointestinal Symptom Rating Scale (GSRS) questionnaire and three stool kits (T0-T1-T2) (Genomix4Life Srl, Baronissi, Italy) to collect fecal samples. The GSRS is a questionnaire used to assess gastrointestinal symptoms in individuals. It is a disease-specific instrument often used in research and clinical settings, particularly for conditions like irritable bowel syndrome and peptic ulcer disease. The GSRS is a validated tool with a 15-item scale, grouped into five symptom clusters: Reflux, Abdominal pain, Indigestion, Diarrhea, and Constipation. The GSRS has a seven-point graded Likert-type scale where 1 represents the absence of any symptoms and 7 represents very troublesome symptoms [17]. Furthermore, all patients were asked to fill out a diary to record any adverse event occurring during the treatment and any missed intake of the prescribed doses. All patients gathered fecal samples during their first morning bowel movement, utilizing the provided scoop. Next, the scoop was placed into a vial filled with preservative liquid. Patients securely closed the lid and kept it at room temperature until they delivered the sample (instructions for shipping were also provided). Stool samples were collected at three different time intervals: baseline (T0), after 4 weeks of daily *L. reuteri* LMG P 27481 intake (T1), and 2 weeks after discontinuation of the probiotic (T2). At the same time, patients filled out the GRSS questionnaire. All patients were instructed to take five drops of *L. reuteri* LMG P 27481 (Reuterin^®^ Noos, Nóos S.r.l., Rome, Italy), 5 × 10^9^ colony-forming units (CFU) dissolved in water, once a day in the morning before breakfast, for 28 days (Figure 1).

The probiotic contains 1 × 10^9^ CFU of *L. reuteri* LMG P 27481 for each drop. During the study period, patients were instructed to store the product in the refrigerator (6 °C). Protocol adherence was verified through the empty vial which needed to be returned by the patients on the day after the end of the treatment, and by directly asking the patients about treatment compliance. If the unused study product was >25% of the recommended dose, the patient was considered noncompliant and exited the statistical evaluation. Patients did not receive any grant for their participation in the study. Patients kept their usual lifestyles and dietary intakes throughout the 4 week-long study period and the 14 days of follow-up. This study was approved by the independent Ethics Committee of San Camilo Forlanini Hospital in Italy (ID 126-2024) and was conducted according to the Declaration of Helsinki.

### 3.1. Microbiota Analysis

Microbiota analysis was performed using a genomix4life kit. Each patient was given three kits numbered T0-T1-T2 for stool collection. Each kit included a container to collect a sample, which was then immersed in the inactivating liquid provided inside the container. The stool samples could be stably transported and store stabilized DNA at ambient temperature for 60 days. At enrollment, a stool sample was obtained at baseline before probiotic ingestion (T0), after 4 weeks of supplementation with the probiotic (test sample T1), and after 2 weeks from the suspension of treatment (test sample T2). All samples were analyzed within 30 days.

The pipeline analysis includes the following steps for processing, analyzing, and interpreting sequencing data: DNA isolation and NGS sequencing were performed at Genomix4Life Srl (Baronissi, Italy). Bacterial DNA was isolated from stool samples using the QIAamp Fast DNA Stool Mini Kit (Qiagen, Hilden, Germany), specifically designed for the rapid and efficient isolation of microbial DNA from fecal material while preserving the representativeness of the intestinal microbiota. The kit combines mechanical and chemical lysis to ensure effective disruption of bacterial cell walls, including those of Gram-positive species, which are typically more resistant. The protocol includes an initial lysis step using InhibitEX buffer (Hilden, Germany), which efficiently removes PCR inhibitors and contaminants commonly present in stool samples, followed by DNA purification on silica-based spin columns via centrifugation. The yield and quality of the extracted DNA were assessed using a NanoDrop spectrophotometer (Thermo Scientific, Waltham, MA, USA) by measuring the A260/280 and A260/230 ratios to estimate sample purity. Double-stranded DNA concentration was further quantified using the Qubit Fluorometer (Invitrogen, Carlsbad, CA, USA). PCR amplification was performed using primers targeting the hypervariable V3–V4 region of the 16S rRNA gene: Forward primer: 5′-CCTACGGGNGGCWGCAG-3′; and reverse primer: 5′-GACTACHVGGGTATCTAATCC-3′. Each PCR reaction was assembled following the 16S Metagenomic Sequencing Library Preparation protocol (Illumina, San Diego, CA, USA). Libraries were quantified using the Qubit Fluorometer (Invitrogen, Carlsbad, CA, USA) and pooled in equimolar amounts to a final concentration of 4 nM, including 25% of the PhiX Control Library (Illumina). Pooled libraries were subjected to cluster generation and sequenced on the Illumina MiSeq platform (Illumina, San Diego, CA, USA) using a 2 × 250 bp paired-end format at a final loading concentration of 10 pM. The raw sequence files (FASTQ format) were subjected to quality control analysis using FastQC v.1.0. Sequence data were analyzed to determine bacterial community composition across taxonomic levels, following standard pipelines for denoising, taxonomic assignment, and quality control [18]. We used the deep sequence of 100,000 reads. Sequence data were processed using the DADA2 pipeline (version 1.26, R environment), which includes quality trimming, denoising, and chimera removal. Amplicon sequence variants (ASVs) were taxonomically assigned against the SILVA reference database (version 138) at 97% sequence similarity. After quality filtering, the mean sequencing depth per sample was 52,000 reads (range 38,000–67,000), ensuring sufficient coverage as confirmed by rarefaction curve analysis.

Alpha diversity was assessed using Shannon and Pielou’s evenness indices, whereas beta diversity was evaluated using Bray–Curtis dissimilarity metrics Intra-individual changes across time points (T0 and T1 and T2) were analyzed using paired Wilcoxon signed-rank tests. Differences in relative abundances at the phylum and genus levels were evaluated with ANCOM and Kruskal–Wallis tests, applying Benjamini–Hochberg correction for multiple comparisons. Statistical significance was set at *p* < 0.05 *.

The algorithms used to derive the reported indices were developed working on a dataset comprising nearly 20,000 human fecal samples processed via 16S rRNA gene sequencing through a combination of statistical modeling, machine learning, and literature-based reference mapping) [19]. All metrics were normalized on a 0–100 scale unless otherwise indicated, allowing for inter-individual comparison across key dimensions of gut microbiota structure and function [20]. The following parameters were computed as the intestinal barrier integrity (evaluated through the analysis of the relative abundance of taxa known to influence mucus layer stability and epithelial tight junctions, such as *Akkermansia muciniphila* and *Bifidobacterium* spp. [21].

In the present study, intestinal barrier integrity and permeability were inferred from microbial signatures associated with mucus layer stability and tight junction regulation, following validated bioinformatic algorithms and previously published references [21,22,23]. Specifically, taxa such as *Akkermansia muciniphila* and *Bifidobacterium* spp. were used as key indicators of mucosal health. These computational indices were generated using the standardized analysis pipeline provided by Genomix4Life Srl (Baronissi, Italy), ensuring methodological consistency across samples.

Results are expressed as a percentage estimate of mucosal protection. Barrier effectiveness: it reflects the protective role of the intestinal mucosa against the translocation of pro-inflammatory and antigenic compounds. This parameter is based on microbial signatures associated with mucus production, epithelial integrity, and tight junction regulation [22]. Dysbiosis index: it is calculated by integrating the relative abundances of microbial taxa with known pro- and anti-inflammatory effects, according to international literature [23]. Higher percentages denote more pronounced dysbiotic profiles. Firmicutes/Bacteroidetes ratio: it is reported as an absolute value and reflects the balance between the two dominant phyla, historically associated with metabolic and inflammatory conditions [24]. Short-chain fatty acids (SCFA): they are indirectly estimated by evaluating the presence of known SCFA-producing bacteria for acetate, propionate, and butyrate [25]. Each compound is quantified individually on a normalized 0–100% scale. Biodiversity index: it is calculated using Pielou’s evenness index, which considers both species richness and the balance of their distribution. Values closer to 1 indicate higher microbial diversity [26]. Gut–brain axis modulation: it is inferred from the presence of microbial taxa involved in tryptophan metabolism and the biosynthesis of neuroactive compounds (e.g., *Faecalibacterium prausnitzii*, *Bifidobacterium adolescentis*, *Coprococcus* spp.) [27]. This composite index estimates both stress sensitivity and neuroactive production potential. Metabolic function: it is assessed through microbial profiles linked to anabolic efficiency and glucose metabolism [28]. Results are presented as normalized percentage scores. Each algorithm integrates deviations from internal reference distributions using percentile-weighted scoring derived from curated scientific literature [29]. The computational framework simultaneously analyzes multiple taxa for each parameter to enhance robustness and reduce signal noise [30]. Algorithmic validation was performed through an ongoing observational in-house program, which monitors clinical utility and consistency across large-scale applications [31]. This continuous validation strategy follows the principles of applied bioinformatics and real-world evidence generation [32,33,34,35].

### 3.2. Statistical Analysis

Statistical analyses were performed using STATA version 14.0 (StataCorp, College Station, TX, USA) and R software (version 4.3.2) with the DADA2 and Phyloseq packages v. 3.6 for microbiota data processing. Continuous variables are expressed as mean ± standard deviation (SD), and categorical variables as absolute values and percentages. For microbiota composition, alpha diversity indices (Shannon, Pielou’s evenness) were computed to assess within-sample diversity, and beta diversity was calculated using Bray–Curtis dissimilarity to evaluate inter-sample variability across time points (T0, T1, T2). Intra-individual changes were analyzed using paired Wilcoxon signed-rank tests. Differential abundance of microbial taxa at the phylum and genus levels was assessed using the Analysis of Composition of Microbiomes (ANCOM) method and Kruskal–Wallis tests, applying the Benjamini–Hochberg correction for multiple comparisons. For clinical outcomes derived from the Gastrointestinal Symptom Rating Scale (GSRS), repeated-measures comparisons among time points were performed using the Friedman test followed by Dunn’s post hoc analysis where appropriate. Statistical significance was set at *p* < 0.05 * (two-tailed).

Microbial community diversity was assessed through alpha diversity indices (Shannon and Pielou’s evenness) to evaluate within-sample heterogeneity, and beta diversity using Bray–Curtis dissimilarity to compare microbial structure across time points (T0, T1, T2). These diversity metrics were calculated within the same bioinformatic workflow described in Section 3.1, following established standards in microbiome literature. Intra-individual differences across time points were analyzed using paired Wilcoxon signed-rank tests, with *p* < 0.05 considered statistically significant.

### 3.3. Sample Size

As a pilot study we enroll a total of 20 patients; this sample size is based on practical considerations regarding the feasibility of recruitment, data collection, and other study procedures. This ample size allows a preliminary assessment of study logistics and potential challenges. Considering this as a pilot study, and due to the absence of well-established effect sizes in the literature, a sample size of 20 patients is expected to provide preliminary data on the primary and secondary outcomes. Although this sample size is unlikely to detect statistically significant differences, it may serve as a starting point for estimating effect sizes and variability for future larger-scale studies.

## 4. Results

**Figure 2:**
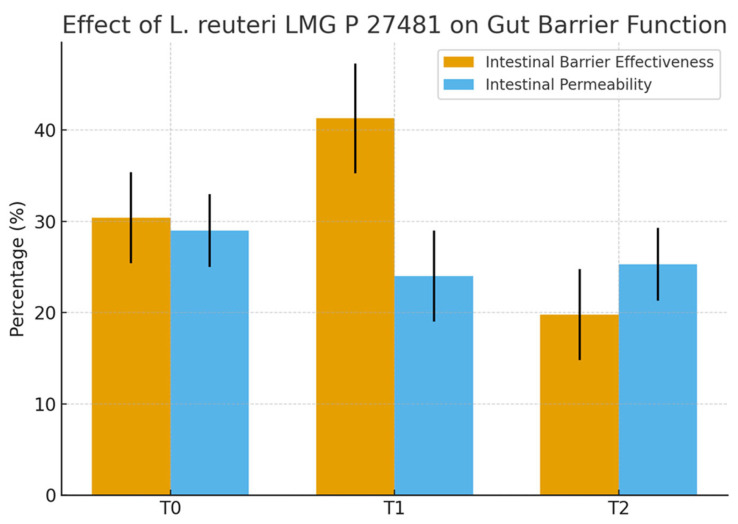
Changes in intestinal barrier effectiveness and intestinal permeability at baseline (T0), after 4 weeks of *L. reuteri* LMG P 27481 supplementation (T1), and after 2 weeks of follow-up (T2). Data represent mean ± SD from 20 healthy subjects (*n* = 20). Statistical comparisons across time points were performed using paired Wilcoxon signed-rank tests.

**Figure 3:**
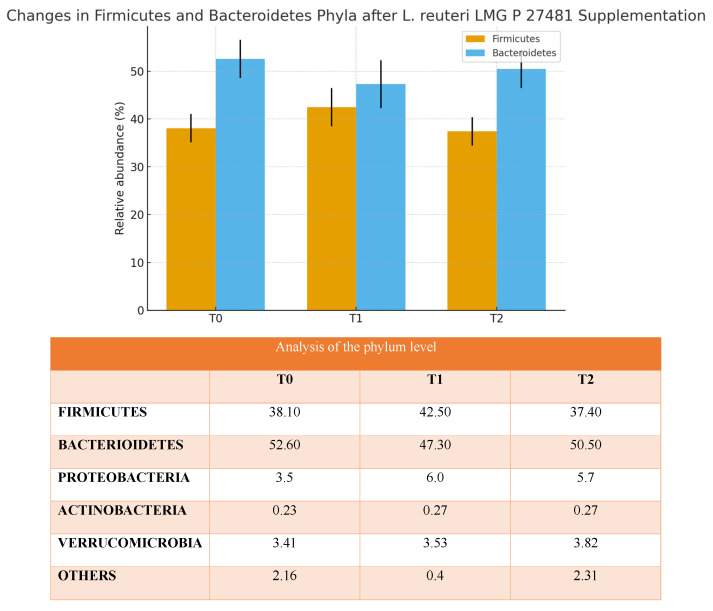
Relative abundance of Firmicutes and Bacteroidetes phyla at baseline (T0), after 4 weeks of *L. reuteri* LMG P 27481 supplementation (T1), and after 2 weeks of follow-up (T2). Data represent mean ± SD from 20 healthy subjects (*n* = 20). Statistical comparisons between time points were performed using paired Wilcoxon signed-rank tests.

**Figure 4:**
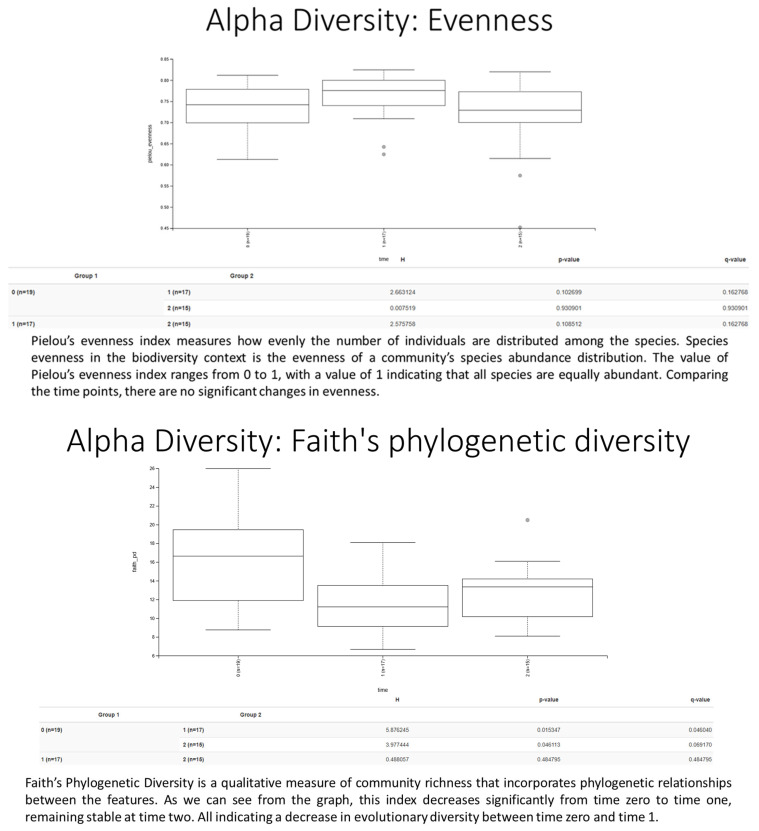
Alpha Diversity.

**Figure 5:**
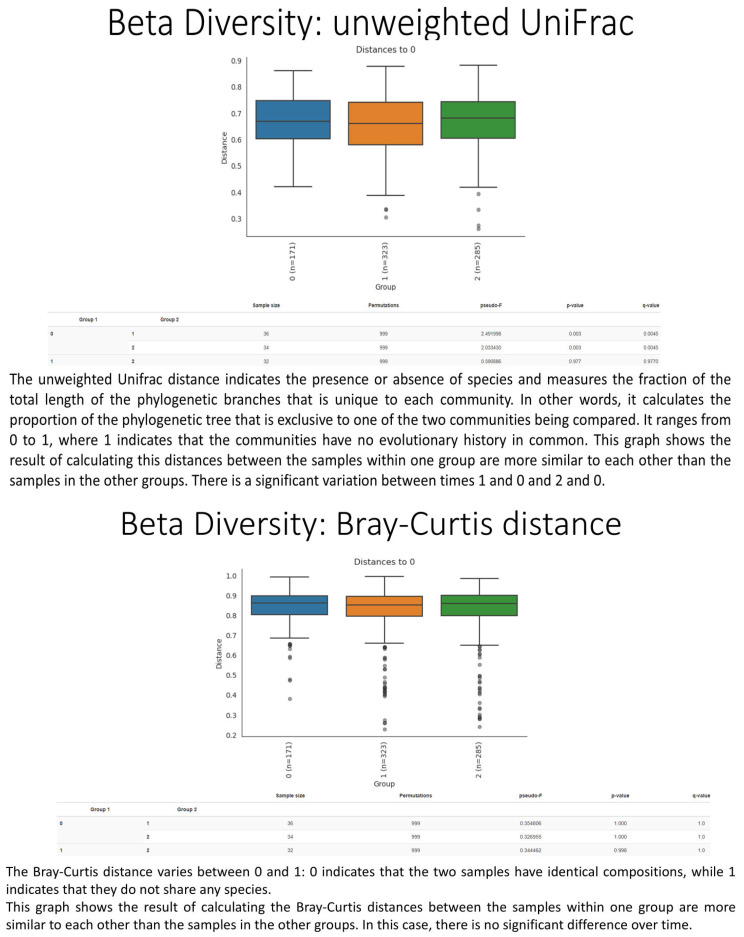
Beta Diversity.

**Figure 6:**
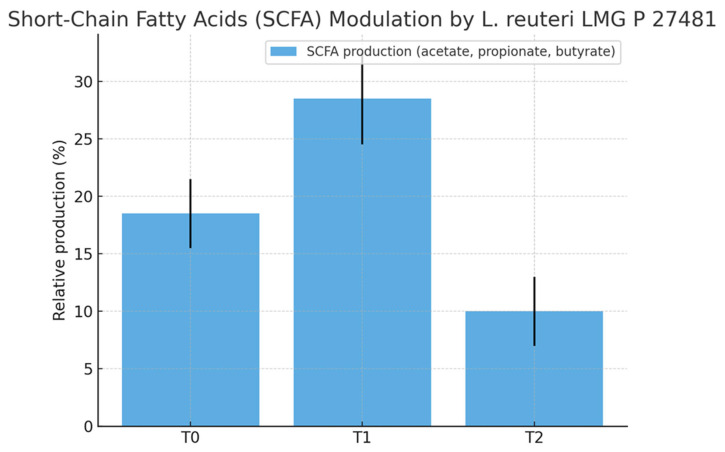
Short-chain fatty acids (SCFA: acetate, propionate, and butyrate) levels at baseline (T0), after 4 weeks of *L. reuteri* LMG P 27481 supplementation (T1), and after 2 weeks of follow-up (T2). Data represent mean ± SD from 20 healthy subjects (*n* = 20). Statistical comparisons among time points were performed using paired Wilcoxon signed-rank tests.

**Figure 7:**
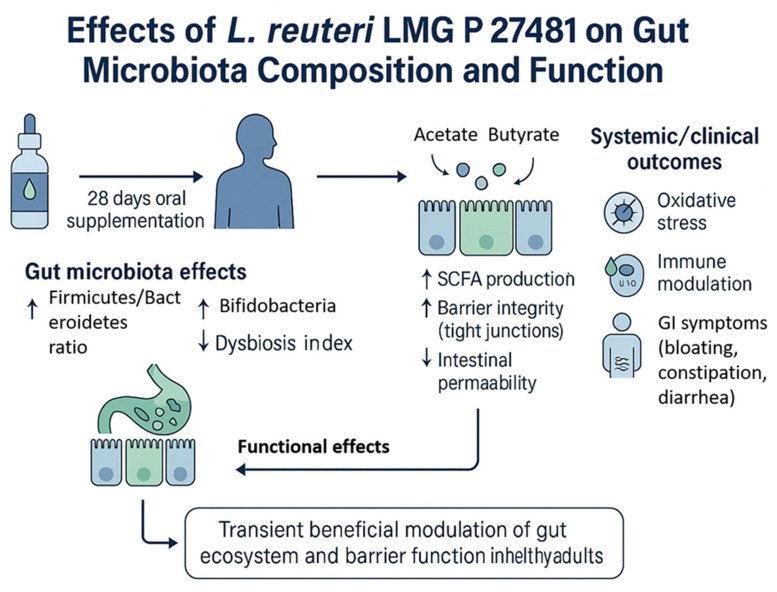
Graphical summary of the effects of *L. reuteri* LMG P 27481 supplementation on gut microbiota composition and function. The schematic illustrates the main mechanisms observed after 28 days of oral administration in healthy adults: an increase in the Firmicutes/Bacteroidetes ratio, enrichment of *Bifidobacterium* spp., and reduction of the dysbiosis index. Functional effects include enhanced SCFA production (acetate, propionate, and butyrate), improved intestinal barrier integrity through tight junction reinforcement, and reduced intestinal permeability. These changes contribute to decreased oxidative stress, improved immune modulation (increased IL-10), and alleviation of gastrointestinal symptoms such as bloating, constipation, and diarrhea. The overall outcome is a transient beneficial modulation of gut ecosystem and barrier function.

A total of 20 healthy adult participants (10 males and 10 females; mean age 34.5 ± 15.5 years) completed the study. All patients adhered to the treatment protocol and returned the probiotic packet at the end of the intervention period. No serious adverse events were reported. Treatment compliance, assessed via patient diaries and returned packet, was achieved in 75% of all cases. None of the subjects received antimicrobial agents during the investigation period. The demographic characteristics of the 20 enrolled patients (age, gender, BMI) are shown in Table 1. At baseline (T0), mean intestinal barrier effectiveness was quite low (30.4%), but markedly increased to 41.3% at T1, followed by a complete loss of improvement at T2 (19.8%). An inverse trend was observed for intestinal permeability, which decreased from 29% at T0 to 24.0% at T1, then rose again to 25.3% at T2. The dysbiosis index improved during treatment, decreasing from 43% at baseline to 39.0% at T1 (Figure 2). However, the improvement was not sustained after probiotic withdrawal, with values returning to 52.0% at T2. Taxonomic analysis at the phylum level revealed a shift in the Firmicutes/Bacteroidetes (F/B) ratio: baseline values were 0.7, increased to 0.9 post-treatment, and returned to 0.74 at follow-up. The relative abundance of Firmicutes increased from 38.1% (T0) to 42.5% (T1), while Bacteroidetes decreased from 52.6% to 47.3%. At T2, both phyla partially reverted toward baseline proportions (Firmicutes 37.4%, Bacteroidetes 50.5%, respectively) (Figure 3). Another important observation was the increase of actinobacteria from 0.3% at enrollment to 0.77%, evidence of an increase of *Bifidobacterium* species. As regards alpha diversity evenness, the value of Pielou’s evenness index ranges from 0 to 1, with a value of 1 indicating that all species are equally abundant. We do not find any significant changes comparing the time points (Figure 4). As regards beta diversity-unweight UniFrac, we found a significant variation between time 1 and 0 and time 2 and 0 (Figure 5). This pattern aligns with transient increases in ecosystem gut complexity during probiotic administration. Regarding microbial functional potential, SCFA production increased from 18.5% at T0 to 28.5% at T1 and returned to 10% at T2 (Figure 6). Exopolysaccharides (EPS), which are long-chain sugar molecules produced by bacteria, fungi, and microalgae, play a crucial role in biofilm formation, and help microbes adhere to surfaces, creating a protective matrix. They show an increase after treatment from 21.2% to 27.5%, but a rapid decrease after 14 days of follow-up to 21.6%. The oxidative stress protection index, which was 35.7% at baseline, rose to 37.7% post-treatment and decreased to 18.8% at follow-up. Similarly, immune modulation potential increased from 24.4% (T0) to 30.3% (T1), but returned to baseline values at 15% (T2). In terms of the gut–brain axis, the stress sensitivity index slightly improved during treatment (decreasing from 35.8% to 27%), but increased again after discontinuation (43.0%). In our study we noted a modulation in tryptophan metabolism, with an increase in levels from 14.6% to 38% during treatment, then a decrease to 29.3% at T2. Furthermore, our study revealed a reduction of trimethylamine N-oxide (TMAO) levels, from 9.4 at enrollment to 8.1 at the end of treatment, and a further reduction at T2 to 6.8, suggesting a beneficial effect of *L. reuteri* LMG P 27481 on this metabolite [33]. Analyzing the data, we noticed that patients who presented a higher intestinal dysbiosis at enrollment (>50%) (6/20 pts) showed a mean negligible intestinal barrier effectiveness at baseline (10.2%), but obtain a marked increased to 42.8% at T1, followed by a complete loss of improvement at T2 (9.8%). A similar but inverse trend was observed for intestinal permeability, which decreased from 30.5% at T0 to 11.0% at T1, then increased again to 13.0% at T2. The dysbiosis index improved during treatment, decreasing from 60.0% at baseline to 49.0% at T1. However, the improvement was not sustained after probiotic withdrawal, with values returning to 61.0% at T2. At enrollment, these kinds of patients presented a relatively low level of Firmicutes and a high level of Bacteroidetes of 15.0% and 81.0%, respectively, but, at the end of *L. reuteri* LMG P 27481 supplementation, had a significant increase of Firmicutes to 40% and a decrease of Bacteroidetes to 51%, achieving a better F/B ratio. Unfortunately, at T2, both phyla partially reverted toward baseline proportions (Firmicutes 17.0%, Bacteroidetes 76.0%). The biodiversity index (Pielou’s evenness) in these patients improved during treatment, rising from 0.10 at baseline to 0.23 at T1, before decreasing to 0.13 at T2. A summary of the effects of *L. reuteri* LMG P 27481 supplementation on gut microbiota composition and function is shown in Figure 7.

### GSRS Questionnaire

As regards the 15-item scale (from 0 to 6), grouped into five symptom clusters: Reflux, Abdominal pain, Indigestion, Diarrhea, and Constipation we do not observe any modification as regards Reflux, while the best results were obtained for Indigestion. Diarrhea went from a mean value of 2 at T0 to 0.27 at T1, and Constipation from 2.6 to T0 to 0.6 T1. The beest results were also obtained for bloating and passing gas, with a significant decrease from 3 to 0.6 and from 3.27 to 1.1, respectively (Table 2). The parameters analyzed included the philum level are shown in Table 3.

## 5. Discussion

This pilot study aimed to investigate the functional impact of the probiotic strain *L. reuteri* LMG P 27481 on the gut microbiota composition and host-microbiota interactions in 20 healthy adults after 28 days of oral supplementation. One of the most striking effects observed was the temporary improvement of the intestinal barrier function, with an increase in the barrier effectiveness index from 30.4% at baseline to 41.3%. This enhancement was accompanied by a decrease in the intestinal permeability index (from 29.0% to 24.0%). These data confirm many previous studies that highlight the fact that *L. reuteri* promotes gut mucosal integrity and reduces bacterial translocation. This is achieved by promoting the production of antimicrobial peptides and tight junction proteins, which help to strengthen the gut barrier. Additionally, *L. reuteri* can modulate the Wnt/β-catenin signaling pathway, which is important for intestinal epithelial regeneration and repair. Some studies (both in vitro and in vivo) showed the capability of *L. reuteri* to lower intestinal permeability and enhance the expression of tight junction proteins like occludins and claudin-3. The post-treatment decline of both indices to near-baseline values suggests that continuous administration might be necessary to maintain these effects over time. Not only a strong gut barrier, but also an optimal balance among gut microbiota members, their microbial diversity, and metabolites are essential for intestinal health. At the end of treatment in our patients, we observed an improvement in the dysbiosis index (43.0% → 39.0%), suggesting a partial rebalancing of the microbial community. However, the return to baseline values at T2 supports the hypothesis that *L. reuteri* temporarily promotes a more balanced ecosystem without reshaping it permanently. Other studies have shown that *L. reuteri* supplementation [36,37,38] can increase bacterial diversity in the gut, especially in cases where the gut microbiota is less diverse, such as in preterm infants [39]. Taxonomic shifts further supported this temporary modulation [40,41,42,43,44,45]. Notably, we observed an increased in Firmicutes/Bacteroidetes (F/B) ratio during treatment This shift was due to an increase in Firmicutes (38.1% → 42.5%) and a decrease of Bacteroidetes (52.6% → 47.3%). Although the clinical significance of the F/B ratio remains debated, some studies associate a higher ratio with improved metabolic efficiency and energy harvesting, while others suggest it may correlate with pro-inflammatory states [46,47,48,49,50]. *L. reuteri* can directly inhibit the growth of pathogenic bacteria such as *H. pylori* or *C. difficile* through the production of reuterin and promote the growth of beneficial bacteria. This can lead to a more balanced and diverse gut microbial community, reducing the overgrowth of harmful bacteria associated with dysbiosis. Moreover, some studies have shown that *L. reuteri* can reduce the abundance of Proteobacteria and increase Firmicutes, often associated with a healthy gut. Both animal and human studies support these results: one animal study performed on scurfy mice with gut microbial dysbiosis shows an increase in the phylum Firmicutes (in particular genera *Lactobacillus*) after oral administration of *L. reuteri* strain (DSM17938), leading to a reduction in multi-organ inflammation [40]. In another study performed in healthy adults, the administration of capsules containing *L. reuteri* NCIMB 30242 for 4 weeks were able to increase the ratio of Firmicutes to Bacteroidetes [42], supporting again our results. Of particular interest is also the increase of Actinobacteria phylum observed at the end of treatment. They contribute to gut health and overall well-being by helping to digest complex carbohydrates, producing beneficial compound like SCFAs, and maintaining a healthy intestinal barrier. Both animal models and human studies showed that *L. reuteri* can increase the abundance of bacteria within the Actinobacteria phylum, particularly the *Bifidobacterium* genus. This effect is likely due to the ability of certain *L. reuteri* strains to produce metabolites that favor the growth and colonization of these beneficial bacteria [43]. The rise in SCFA production that we observed in our population from 18.5% to 28.5% at the end of treatment may have been caused by the increase in *Lactobacillus* and *bifidobacteria* level. Butyrate and propionate, two SCFAs, are essential for gut health maintenance, intestinal lining integrity preservation, gut motility regulation, immune response modulation, and general gastrointestinal support. SCFAs can upregulate EPS biosynthetic genes expression in both *L. reuteri* and other resident gut bacteria. After 28 days supplementation with *L. reuteri* we obtain a modulation of EPS-producing taxa, which increased from 21.2% → 27.5% and then dropped again to 21.6 after withdrawal. EPS directly influences the bacterial interactions with the hosts both in modulation of immune responses and bacterial adhesion to the gastro-intestinal epithelium. It is also hypothesized that some health benefits of probiotics are linked to specific EPS structures that probiotics produce [44]. Previous studies suggest that *L. reuteri* produces EPS that capture cholesterol and prevent its absorption, and furthermore produce an enzymatic complex, called bile salt hydrolase (BSH), that reduces intestinal absorption of cholesterol from food, which will then be eliminated through feces. The gut microbiome can impact cardiovascular health through several mechanisms, including the production of metabolites like trimethylamine-N-oxide (TMAO), short-chain fatty acids, and bile acids, as well as through modulating inflammatory pathways. The gut microbiota in fact converts dietary nutrients such as choline and carnitine into trimethylamine (TMA), which is then transformed into TMAO by the liver. Some studies have linked high TMAO levels in the blood to an increased risk of heart attacks, strokes, and other cardiovascular diseases with a dose dependent on the relationship between TMAO concentration and cardiovascular risk. Some studies suggest that certain probiotics, like *L. rhamnosus* GG, can reduce TMAO levels. The specific impact of *L. reuteri* on TMAO is not well-established, but in our study, we highlight the beneficial effect of *L. reuteri* LMG P 27481 supplementation on this metabolite with a reduction level from 9.4 at enrollment to 8.1 at the end of treatment. Functionally, *L. reuteri* appeared to exert beneficial effects on oxidative stress regulation and immune modulation, with respective indices rising from 35.7% to 37.7% and from 24.4% to 30.3% respectively, during supplementation. However, these effects were also lost at follow-up, reinforcing the hypothesis of the strain’s transient action and the potential need for continuous administration or combination with other microbiota-supporting strategies (e.g., prebiotics or dietary fiber). *L. reuteri* exhibits multiple adaptive mechanisms to reduce oxidative stress and withstand reactive oxygen species (ROS). It employs enzymatic antioxidant systems and increases gut-derived propionate, which activates the AMPK pathway, and finally produces metabolites, such as histamine, indole derivatives, and reuterin, which suppress inflammatory responses, indirectly reducing ROS generated during inflammation. To support our results, a study showed that in an animal model, *L. reuteri* DSM17938 can offer protection against necrotizing enterocolitis by reducing oxidative stress and increasing the antioxidant capacity of the intestinal tissue [45], leading to suppressed intestinal inflammation. Finally, in our study, the gut–brain axis appeared to be modulated as well, with a decrease of the stress sensitivity index from 35.8% to 27.0% during treatment. According to Dimidi et al. [46], *L. reuteri* modulate the gut movements via interaction with the gut–brain axis. A study by Liang Zhag et al. [47] demonstrated that in a stressed mouse model, *Limosilactobacillus reuteri* aids in lowering anxiety and intestinal problems. In our study, we observed a change in tryptophan concentration, which increased from 14.6% to 38% during treatment and then decreased to 29.3% at T2. Tryptophan is one of the neurotransmitters essential for gastrointestinal and brain preprocess. It is an essential amino acid metabolized in the gut by bacteria, resulting in a range of metabolites that impact human health and homeostasis. These metabolites have an impact on the brain–gut axis and play a key role in regulating immune responses and the intestinal barrier. Tryptophan is also used by the host to produce melatonin and serotonin, which are essential for mood regulation, sleep, and gastrointestinal motility. These findings support previous research on the psychobiotic properties of these species and suggest that *L. reuteri* may influence neuroactive circuits via metabolites derived from microbes. The fact that this measure returned to baseline values after treatment cessation once again demonstrated the durability of the observed effects. As regards clinical symptoms, *L. reuteri* was effective on abdominal pain, bloating, and alteration of evacuation after 28-day oral administration. Both diarrhea and constipation are the consequence of dysbiosis, which leads to a dysregulation of the complex interaction between microbe communities and host immune systems. Many trials confirm a beneficial effect of *L. reuteri* supplementation on gut bowel movements [48]. *L. reuteri* supplementation is beneficial and safe for treating and preventing diarrhea, as it lessens the severity and length of symptoms. On the other hand, *L. reuteri* helped both adults and children with persistent constipation have better bowel movements. According to Kubota et al. [49], when *L. reuteri* was given twice daily for four weeks to children who had chronic constipation, the gut microbiota’s composition changed (fewer Clostridiales genera, including Oscillospira, Megasphaera, and Ruminococcus), intestinal motility was improved, and transit time increased. These changes were significant after the fourth week of administration. Our group highlighted that the administration of *L. reuteri* twice a day for four weeks was effective in reducing methane production by *Methanobrevibacter smithii*, with an increase in bowel movements and a subsequent improvement of constipation. In conclusion, *L. reuteri* influences gas production during digestion through several important processes, which contributes to the improvement in bloating and gas production that we observed. Initially, it inhibits the proliferation of gas-producing bacteria, which may lead to excessive fermentation and gas production. Additionally, it restricts the supply of food for gas-producing microorganisms, which further lessens gas production and related symptoms. Additionally, *L. reuteri* caused the breakdown of carbohydrates, reducing the amount of undigested carbohydrates that would normally make it to the colon, where gas-producing bacteria would ferment them. Finally, the synthesis of SCFAs, which support colon cells, may reduce gas generation by promoting healthy fermentation activities.

However, we acknowledge that in the present pilot study, SCFA assessment was performed indirectly through metagenomic inference of bacterial taxa known to produce acetate, propionate, and butyrate. This approach, although widely applied in exploratory microbiome studies, does not replace direct biochemical quantification. Future studies should therefore include targeted metabolomic analyses to directly measure individual SCFA concentrations and confirm these preliminary findings.

Lastly, it is significant that in a small subgroup of patients with severe dysbiosis, we obtained the best outcomes after treatment with *L. reuteri* LMG P 27481. We discovered that whereas intestinal permeability was dramatically decreased in these patients, SCFA synthesis and the gut barrier were significantly increased. These findings suggest that probiotics work best in cases of severe dysbiosis. Although many probiotics have beneficial clinical health effects, little is known about how they change gut microbiota. According to a systematic review by Kristensen et al. [34], probiotic supplementation in seven randomized controlled trials did not significantly alter the composition and functions of the gut microbiota in healthy individuals. Similarly, just four out of 29 studies (20%) examined in a different study on healthy individuals by McFarland et al. [35] indicated that probiotics have a limited ability to change the gut flora. The possible explanation could be secondary to the different dosages used, which range from 1 × 10^8^ to 1012 CFU; the use of a single strain versus a combination of strains; the single-day versus twice-daily administration; or alternative modes of administration and treatment duration. Furthermore, numerous studies were conducted on patients with a variety of gastrointestinal illnesses rather than on healthy individuals. Lastly, the diverse techniques for evaluating fecal microbiota must be taken into account. Despite the limited sample size and the short observational period, our data reveals a set of relevant microbial and functional modulations induced by *L. reuteri* LMG P 27481, even if they appear to be transient and reversible upon treatment cessation. The present study, while offering interesting preliminary insights into the effects of *L. reuteri* LMG P 27481 on gut microbiota, is subject to some limitations that must be considered. First, the sample size was very small, including only 20 participants, which limits the statistical power and generalizability of the findings. The follow-up was limited to only 14 days after treatment withdrawal, which does not allow assessment of long-term persistence of the observed effects. Taken together, these limitations indicate that the findings should be interpreted with caution.

## 6. Conclusions

The present pilot study suggests that oral supplementation with *L. reuteri* LMG P 27481 for 28 days in healthy adults induces significant, albeit transient, improvements in microbial composition, intestinal barrier integrity, metabolic output (SCFA and exopolysaccharides), oxidative stress regulation, immune modulation, and gut–brain communication. These effects appeared to reverse within two weeks of treatment cessation, suggesting that long-term or repeated administration may be required to achieve sustained benefits. Despite the limitations inherent to the pilot design (including the small sample size, lack of a placebo control arm, and short follow-up) the findings highlight the promising functional potential of this *L. reuteri* strain. In conclusion, analyzing the gut microbiota after probiotic intake provides valuable insights into the mechanisms by which probiotics exert their beneficial effects. Understanding the complex interplay between probiotics and the gut microbiome is crucial for optimizing probiotic interventions and promoting gut health. We observed that *L. reuteri* supplementation could modified the gut microbiota balance and should be investigated in patients with dysbiosis. This study is a pilot exploration and other studies (including larger sample sizes, randomized placebo-controlled designs, longer follow-up periods, and inclusion of objective biomarkers) are needed to confirm and extend these preliminary results.

The most common findings observed in gut microbiota analysis after intake with probiotics include: (a) modest changes in overall composition: probiotics may not drastically alter the overall composition of the gut microbiota, but they can increase the abundance of specific beneficial bacteria, such as *Bifidobacteria* and *Lactobacilli*; (b) increased diversity: probiotics can contribute to a more diverse and balanced gut microbiota, which is often associated with better health outcomes; and (c) changes in microbial metabolites: probiotics can influence the production of SCFAs and other metabolites, such as TMAO, which play a key role in the gut health and overall well-being of individuals.

## Figures and Tables

**Table 1:** Demographic characteristics.

**Total number of patients**	**20**
**Male**	**10**
**Female**	**10**
**Mean age (yrs) mean ± SD**	**34.5 ± 15.5**
**BMI mean ± SD**	**23.7 ± 3.9**
**Physical activity**	
**Light N/total (%)**	**8/20 (40)**
**Moderate N/total (%)**	**10/20 (50)**
**Vigorous N/total (%)**	**2/20 (10)**
**Type of alimentation**	
**Omnivores N/total (%)**	**19/20 (95)**
**Vegetarian N/total (%)**	**1/20 (5)**
**Smoke N/total (%)**	**12/20 (60)**
**Lactose intolerance N/total (%)**	**6/20 (30)**

**Table 2:** GSRS questionnaire baseline (T0), after 4 weeks of daily *L. reuteri* LMG P 27481 intake (T1), and 2 weeks after discontinuation of the probiotic (T2). *p* < 0.05 *: significant value.

Mean Value	T0	T1	T2	*p*
**Abdominal pain**	**0.91 ± 0.21**	**0.92 ± 0.11**	**0.83 ± 0.22**	0.113
**Heartburn**	**0.27 ± 0.02**	**0.27 ± 0.03**	**0.27 ± 0.05**	0.214
**Acid reflux**	**0.27 ± 0.02**	**0.27 ± 0.01**	**0.27 ± 0.02**	0.121
**Hunger pangs**	**0.0**	**0.0**	**0.0**	0.0
**Nausea**	**0.43 ± 0.21**	**0.42 ± 0.31**	**0.41 ± 0.30**	0.101
**Rumbling**	**0.80 ± 0.41**	**0.71 ± 0.40**	**0.81 ± 0.22**	0.322
**Bloating**	**3.02 ± 2.51**	**0.61 ± 1.40**	**1.03 ± 0.81**	<0.05 *
**Belching**	**0.45 ± 0.20**	**0.32 ± 0.11**	**0.45 ± 0.21**	0.124
**Passing gas**	**3.27 ± 2.42**	**1.10 ± 0.41**	**1.12 ± 0.61**	<0.05 *
**Constipation**	**2.62 ± 1.41**	**0.62 ± 0.41**	**0.91 ± 0.53**	<0.05 *
**Diarrhea**	**2.01 ± 0.41**	**0.27 ± 0.12**	**0.45 ± 0.23**	<0.05 *
**Loose stool**	**0.18 ± 0.10**	**0.0**	**0.0**	0.112
**Hard stool**	**1.45 ± 0.41**	**1.41 ± 0.62**	**1.31 ± 0.72**	0.232
**Urgent bowel movement**	**1.81 ± 0.52**	**1.21 ± 0.31**	**1.33 ± 0.64**	0.225
**Sensation of not completely emptying bowel**	**0.27 ± 0.13**	**0.0**	**0.0**	0.112

**Table 3:** Parameters analyzed, included the ***phylum level***.

Parameters Analysed in the Study
Intestinal barrier integrity
Barrier effectiveness
Dysbiosis index
Firmicutes/Bacteroidetes ratio
Biodiversity Index
Shorty chain fatty acid
Stress sensitivity
Exopolysaccharides (EPS)
Oxidative stress protection index
Stress sensitivity
Tryptophan metabolites
TMAO metabolites

## Data Availability

The original contributions presented in this study are included in the article. Further inquiries can be directed to the corresponding author.
